# Cellulose Membranes Embedded with Gold–Silver Bimetallic Nanoparticles for the Efficient Reduction of 4-Nitrophenol

**DOI:** 10.1021/acsomega.4c09636

**Published:** 2025-04-07

**Authors:** Maíra Vasconcelos de Carvalho, João Henrique G. Lago, Samar Hajjar-Garreau, Fernanda F. Camilo, Larissa V. F. Oliveira

**Affiliations:** † Chemistry Department, Institute of Environmental, Chemical and Pharmaceutical Sciences, Federal University of São Paulo, SP-09913-030 Diadema, Brazil; ‡ Center of Natural Sciences and Humanities, Federal University of ABC, SP-09210-580 Santo Andre, Brazil; § Institut de Science des Matériaux de Mulhouse, CNRS UMR 7361, Université de Haute-Alsace, F-68100 Mulhouse, France; ∥ Université de Strasbourg, F-67081 Strasbourg, France

## Abstract

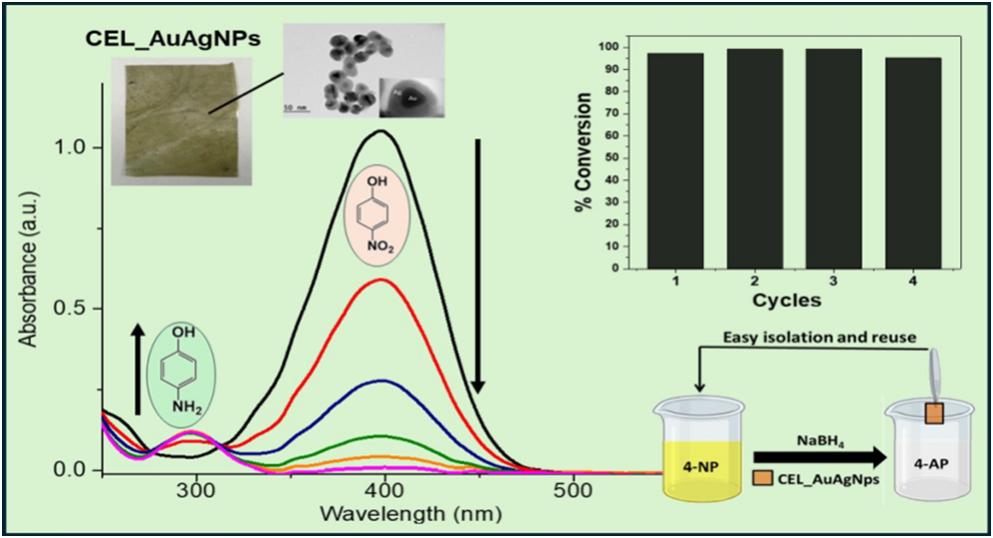

Bimetallic nanoparticles (BNPs) have attracted much attention recently due to their improved properties compared to monometallic ones. Gold and silver nanoparticles (AuAgNPs) are among the most studied BNPs. Using these particles as powder or dispersion has drawbacks such as ease of aggregation and difficulty separating and recovering from the reaction medium. Therefore, immobilizing these nanoparticles in polymeric matrices, such as cellulose, is appealing. In this context, the present work focused on preparing unmodified cellulose membranes containing AuAgNPs for use as heterogeneous catalysts in reducing the pollutant 4-nitrophenol. Incorporating these nanoparticles into cellulose membranes represents a significant advancement in heterogeneous catalysis. In addition to being eco-friendly, cellulose membranes offer ease of handling and the potential for reusability, which are crucial factors in catalysis. The nanoparticles were prepared in an aqueous medium from the seeded growth of a silver shell around AuNP seeds. Images recorded by transmission electron microscopy showed that the particles have diameters smaller than 100 nm and are core–shell type. The cellulose membrane was prepared by dissolving microcrystalline cellulose in an ionic liquid, followed by a regeneration process using water. Next, the bimetallic nanoparticles were incorporated into the cellulose membrane. The analyses revealed that the membranes contain bimetallic nanoparticles homogeneously distributed in the matrix, and the inductively coupled plasma-optical emission spectroscopy (ICP-OES) showed that the membrane has 0.339 wt % in silver and 0.069% in gold. The membranes produced were efficient heterogeneous catalysts in reducing 4-nitrophenol, used for at least four cycles without loss of efficiency. This material can be easily isolated from the reaction medium, avoiding centrifugation or filtration processes for reuse. This study represents the first use of AuAgNPs supported on nonmodified cellulose membranes as heterogeneous catalysts, marking an advancement in catalysis and material science.

## 1 Introduction

Bimetallic nanoparticles have attracted significant interest in recent years due to their outstanding optical, electronic, chemical, and catalytic properties. These characteristics are distinct and often superior to those of their monometallic counterparts, arising from synergistic effects.^1−3^ Among bimetallic nanoparticles, gold nanoparticles (AuNPs) and silver nanoparticles (AgNPs) have been extensively researched in various application areas such as catalysis, biosensors, photothermal therapy, and surface-enhanced Raman scattering (SERS).^4−10^ Their intrinsic properties include high chemical stability, tunable optical features, biocompatibility, and effective catalytic activity. Moreover, their similar lattice constants and face-centered cubic crystal structures facilitate the combination of these metals.^11^

However, a significant challenge in using nanoparticles in dispersion or powder forms is their natural tendency to aggregate due to high surface energy, which can reduce or completely lose their unique properties.^12−14^ One approach to circumventing this issue is embedding the nanoparticles within various matrices. This helps prevent aggregation and enhances the recyclability and ease of material removal from the medium. These features are essential when the goal is to use these materials as heterogeneous catalysts. Various materials, including metal oxides, graphene, carbon nanotubes, polymers, and biomaterials, have supported AuAg bimetallic nanoparticles.^15−19^ Cellulose, a natural biopolymer, shows considerable promise as a nanoparticle-support membrane. Its advantages include affordability, abundance, biodegradability, and nontoxicity.^20−22^

While extensive research has been conducted on embedding gold and silver bimetallic nanoparticles in various matrices to mitigate aggregation, studies focusing on immobilization on unmodified cellulose are still scarce. Notably, most existing studies have utilized cellulose in forms such as paper, powder, or fibers rather than membranes.^23−27^

Building upon the recognized potential of unmodified cellulose for immobilizing gold and silver bimetallic nanoparticles, several recent pioneering studies have achieved significant advances in this area. These research efforts affirm the viability of utilizing cellulose in various forms and highlight the enhanced attributes and practical applications of bimetallic nanoparticle-cellulose composites. For example, Tsai and collaborators (2017)^27^ prepared an antibacterial cellulose paper by immobilizing AuAgNPs onto a cellulose paper by heat treatment. The results indicated that this cellulose paper containing gold–silver bimetallic nanoparticles has the potential to be used as an antimicrobial product. Eman and colleagues (2017)^26^ deposited AuAgNPs onto a cellulose solid support (CSS). This cellulosic powder, extracted naturally from plants, was adorned with both bimetallic and monometallic nanoparticles for comparative purposes. The materials demonstrated robust catalytic activity for 4-nitroaniline reduction, with bimetallic particles showing higher catalytic rates than their monometallic counterparts. Asgari and colleagues (2020)^24^ prepared a nanocomposite based on nanofibrillar cellulose (NFC) coated with gold–silver nanoparticles and used it as a SERS substrate for detecting pesticides in lettuce, demonstrating an excellent detection performance. These studies involve the use of nonmodified cellulose primarily in fiber and powder forms, highlighting a gap in research regarding the use of cellulose in membrane form.

Employing cellulose membranes could offer significant advantages, including mechanical strength, homogeneous dispersion of the nanoparticles, and insolubility in water and other solvents, which are critical properties for their use as heterogeneous catalysts.^28−30^ Furthermore, the preference for natural cellulose over its derivatives or other modified biopolymers is compelling. Modification often adds complexity, raises potential environmental concerns, and may undermine the inherent benefits of the natural material, like its biodegradability and nontoxicity.

The development of cellulose membranes incorporating AuAgNPs is relatively recent, and only a few studies have delved into this topic.^31,32^ For instance, Esquivel-Peña and colleagues (2020)^31^ prepared a hybrid material with gold and silver nanoparticles supported on a cellulose nanofibers membrane modified with borate ions. This modification aimed to reduce the water solubility of the membranes by cross-linking the cellulose fibers with borate ions, and the resulting membranes were utilized as catalysts for the reduction of 4-nitrophenol. Hu and team (2020)^32^ developed a nanocomposite using a cellulose membrane decorated with Ag-AuNPs. Their approach involved dissolving cellulose in a NaOH/urea system and using it as a metal-reducing agent. The solution was then placed into a liquid film on a plate and solidified in glycol to produce a regenerated cellulose nanocomposite embedded with bimetallic AuAgNPs. While this material demonstrated significant antibacterial activity against Gram-positive and Gram-negative bacteria, its catalytic activity was not explored.

Our study presents a more straightforward and efficient method for fabricating free-standing cellulose membranes containing AuAgNPs. Contrasting with previous studies, our approach omits steps to modify the cellulose. We also explore their application as catalysts in reducing the environmental pollutant 4-nitrophenol. A notable advantage of our membranes is the straightforward isolation of the catalytic material from the reaction medium. This feature simplifies the recovery and enhances the recyclability of the catalyst, offering a practical and environmentally friendly solution in catalytic processes. This study is the first to explore using a nonmodified cellulose membrane containing bimetallic gold and silver nanoparticles as a heterogeneous catalyst, filling a notable gap in current research.

## 2 Experimental Section

### 2.1 General Procedures

Gold(III) chloride trihydrate (HAuCl_4_·3H_2_O, 99.9%), silver nitrate (99%), sodium citrate (99%), ascorbic acid (99%), 4-nitrophenol (99%), sodium borohydride (98%) and microcrystalline cellulose (powder, 51 μm particle size) were purchased from Sigma-Aldrich and used without any further purification. The ionic liquid 1-butyl-3-methylimidazolium chloride (BMImCl) was prepared following a procedure already described in the literature.^33^

Electronic absorption spectra in the Ultraviolet–visible (UV–vis) region were recorded in an Ocean Optics spectrophotometer model USB 4000. The spectra of liquid samples were acquired in absorbance mode, using a quartz cuvette with 1.00 cm of optical path. The membranes, with a thickness of 0.01 mm, were analyzed in reflectance mode using a custom-designed sample holder. Fourier-transformed infrared absorption spectra (FTIR) were obtained on a Cary 630 spectrometer from Agilent using an attenuated total reflection (ATR) accessory of 650–4000 cm^–1^ with 4.0 cm^–1^ resolution. Inductively coupled plasma-optical emission spectroscopy (ICP-OES) analysis was carried out in an Arcos model from Spectra using argon plasma equipped with a solid-state detector of the charge-coupled device type. Samples were digested in a DigiPrep digester block model from SCP Science. X-ray diffractograms (XRD) were registered using a D8 Advance diffractometer from Bruker with Cu Kα radiation (0.1542 nm) operating at 40 keV and 40 mA. Dynamic light scattering analysis (DLS) and the Zeta potential of the samples were determined using a Malvern Zetasizer Nano instrument. Analyses were made in quintuplicate. Transmission electron microscopy (TEM) with Energy-dispersive X-ray spectroscopy (EDX) line-scan analysis was performed on a JEM 2100 microscope from Jeol. A single drop of the samples was added onto a carbon-coated Cu microgrid and allowed to dry at room temperature. Scanning electron microscopy (SEM) images were recorded on a JSM-7401F from Jeol. Samples were carbon-coated before the analysis. Thermogravimetric analyses (TG) were obtained in a Discovery SDT 650 from TA Instruments. Samples were placed in alumina crucibles under a synthetic air atmosphere with a flow of 100 mL·min^–1^ and a heating rate of 10 °C·min^–1^ from 25 to 1000 °C. X-ray photoelectron spectroscopy (XPS) measurements were conducted using a Thermo Scientific K-α spectrometer with an aluminum monochromator and Al Kα radiation (1486.6 eV). Spectra were fitted with Gaussian–Lorentzian component profiles using CasaXPS software 2.3.18 Ltd., Teignmouth, U.K.

### 2.2 Methods

#### 2.2.1 Synthesis of Bimetallic Gold–Silver Nanoparticles

First, gold nanoparticles (AuNPs) were prepared following a procedure described in the literature.^34^ Then, the bimetallic gold–silver nanoparticles were prepared based on the seeded growing method.^35^ 5.96 mL of the as-synthesized gold nanoparticles (179 mg L^–1^), 4.00 mL of sodium citrate (38.8 mM), and 120 mL of deionized water were stirred for 10 min. Then, 1.60 mL (100 mM) of ascorbic acid followed by 4.14 mL of silver nitrate (10.0 mM) were added dropwise into the system, which was kept under stirring for 30 min at room temperature. This sample was denoted as AuAgNPs.

#### 2.2.2 Preparation of Cellulose Membranes Decorated with the Bimetallic Nanoparticles

Microcrystalline cellulose was dissolved in the ionic liquid BMImCl at a concentration of 5.00 wt % under magnetic stirring and heating at 60 °C for 24 h. Subsequently, 5.00 g of cellulose dispersion was poured into a Petri dish and spread using a spin-coater (500 rpm for 40 s). After that, water was added to the Petri dish to regenerate the cellulose as a membrane. This membrane was thoroughly washed with water to remove residual ionic liquid. Afterward, 100 mL of the AuAgNPs dispersion was added to the Petri dish containing the wet cellulose membrane. To monitor the impregnation process of the AuAgNPs dispersion into the cellulose membrane, aliquots of the dispersion were periodically evaluated using UV–vis spectroscopy, focusing on the Surface Plasmon Resonance (SPR) band of the nanoparticles. After completing this process, the membrane was washed with water and dried at room temperature for 5 days. This sample was designated as CEL_AuAgNPs.

#### 2.2.3 Catalytic Reduction Experiments

The catalytic tests were carried out by adding a square piece of the membrane CEL_AuAgNPs (approximately 1.50 × 1.50 cm; 10.0 mg) into a mixture containing 5.00 mL of 4-nitrophenol (3.00 mM) and 5.00 mL of NaBH_4_ (300 mM). The reaction proceeded with stirring at room temperature. Over time, aliquots were taken from the reaction mixture and analyzed using UV–vis spectroscopy to monitor the progress of the catalytic reduction. After the end of the test, the catalyst was removed from the solution using a tweezer, washed with distilled water, and reused in a new catalytic reduction experiment.

## 3 Results and Discussion

This study presents a novel method for preparing a heterogeneous catalyst using a cellulose membrane embedded with bimetallic gold–silver nanoparticles (CEL_AuAgNPs). These nanoparticles were synthesized via the seeded growth method, where gold nanoparticles served as seeds for forming a silver shell by reducing silver salt.^35^

**Figure 1:**
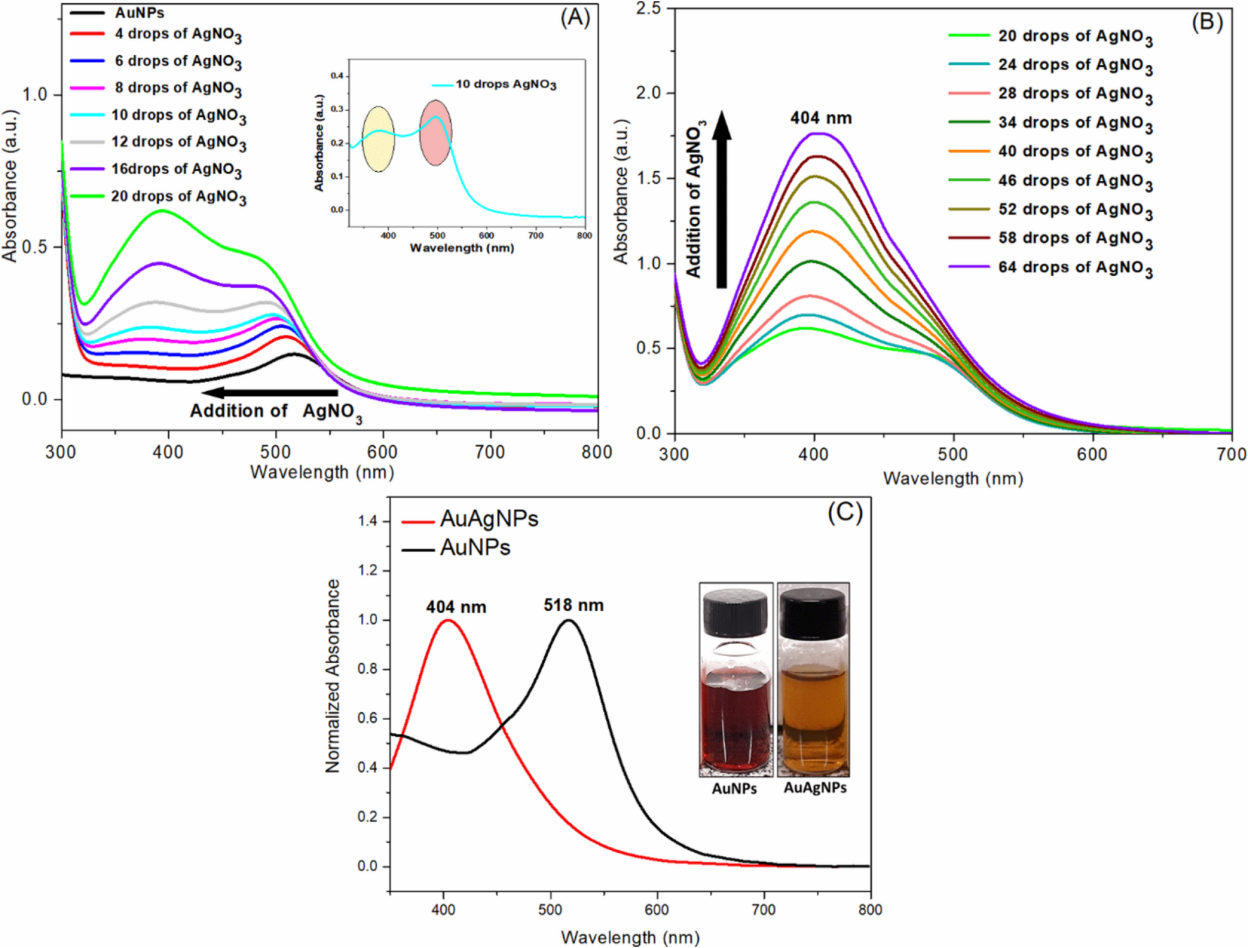
(A and B) UV–vis spectra of the reaction medium during AuAgNPs formation in different stages, and (C) Normalized UV–vis spectra of AuNPs and AuAgNPs dispersions. Inset: Photographs of the nanoparticle dispersions.

Spherical gold nanoparticles (AuNPs) with diameters between 10 and 20 nm were initially synthesized using the Turkevich method (Figure S1). This process involved reducing Au^3+^ to Au^0^ using sodium citrate as a reducing and stabilizing agent at boiling temperature.^36^ Subsequently, these AuNPs were combined with aqueous silver nitrate and ascorbic acid solutions, acting as the reducing agent for silver shell formation. During this reaction, the solution’s color shifted from red to light orange, signifying the growth of the silver shell around the gold core (inset Figure 1C).^35^ This formation of the silver shell was continuously monitored *in situ* via Ultraviolet–visible (UV–vis) spectroscopy by adding controlled amounts of AgNO_3_ dropwise to the solution. The UV–vis spectra in Figure 1A depict the solution after the addition of small quantities of silver salt. The original spectrum of the AuNPs dispersion (black curve) exhibits a band at 520 nm, attributed to the gold nanoparticles’ localized surface plasmon resonance (LSPR).^37^ Upon the addition of the silver salt, this peak shifts to a shorter wavelength, indicating alterations on the surface of the AuNPs. With further additions, a new absorption band emerges around 400 nm, characteristic of silver nanoparticles’ surface plasmon resonance (SPR) (AgNPs).^38^ Notably, after adding a specific volume of AgNO_3_, the spectrum revealed the simultaneous presence of both gold and silver SPR bands (see Figure 1A inset), suggesting the formation of a core–shell nanostructure.^7,39^ As more silver salt was added (Figure 1B), the peak around 400 nm became more prominent in the spectrum due to the increasing deposition of silver onto the gold core. Finally, as observed in Figure 1C (red curve), a single absorbance band at approximately 404 nm dominated, corresponding to the SPR of silver nanoparticles. This observation indicates the development of a thicker AgNP shell rather than the loss of the core–shell structure.^4,9^

**Figure 2:**
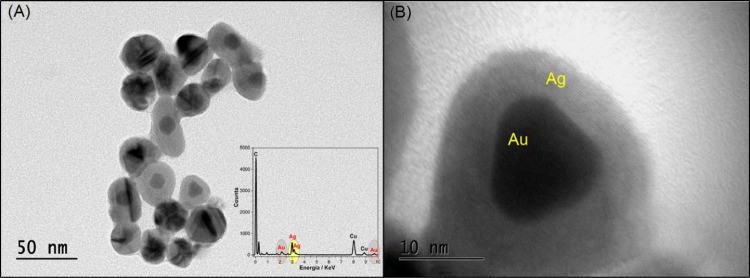
TEM image (A), inset EDX spectrum*, and HRTEM image (B) of the AuAgNPs dispersion. * Cu element on the EDX spectrum is from the grid used in the analysis.

The size and morphology of the AuAgNPs were assessed using transmission electron microscopy (TEM). The particles are predominantly spherical, with diameters ranging from 20 to 30 nm, as shown in Figure 2A. High-transmission electron microscopy (HTEM) imaging (Figure 2B) reveals the core–shell structure of the particles, characterized by a high-contrast gold (Au) core surrounded by a lower-contrast, thicker silver (Ag) shell. Energy-dispersive X-ray Spectroscopy (EDX) analysis, depicted in the inset of Figure 2A, confirms the formation of AuAgNPs. This is evidenced by peaks at 2.98 and 3.15 eV, corresponding to metallic silver, and peaks at 2.12 and 9.71 eV, attributed to gold. The simultaneous detection of both metals in the nanoparticles also indicates the formation of bimetallic AuAgNPs, supporting the findings from the UV–vis spectroscopic analysis.^40^ Additionally, EDX line-scan analysis (Figure S2) further confirmed the core–shell architecture of the nanoparticles. The line scan revealed a strong gold signal at the nanoparticle’s core, consistent with the presence of an Au core. Furthermore, the silver signal becomes increasingly prominent beyond approximately 70 nm, confirming the presence of a silver shell. The delayed prominence of the silver signal can be explained by variations in shell thickness. The silver signal becomes more distinct as the electron beam encounters thicker or denser regions of the shell. Besides, the orientation of the nanoparticle during the line scan may influence the relative visibility of the core and shell. In areas where the silver shell is thinner or overlaps minimally with the gold core, the gold signal appears more pronounced.

The Zeta potential of the AuAgNPs dispersion was measured at −44.5 ± 1.39 mV, indicating a high degree of colloidal dispersion stability due to the favorably negative value. Dynamic Light Scattering (DLS) analysis (Figure S3) revealed a bimodal size distribution within the nanoscopic range. The average hydrodynamic diameters were 3.36 ± 0.26 and 50.98 ± 0.24 nm. Notably, the hydrodynamic diameter is not a direct measure of the particle size itself but rather represents the size of the particle in solution, including surrounding species like solvent molecules and ions. This explains the observed larger sizes compared to those in the TEM images. The Polydispersity Index (PDI) was calculated to be 0.309 ± 0.003, suggesting that the AuAgNPs dispersion contains a predominantly monodisperse particle population. PdI values estimate the size uniformity within a sample; values above 0.7 suggest high polydispersity, whereas values below 0.3 indicate a monodisperse-sized dispersion.^41^

**Figure 3:**
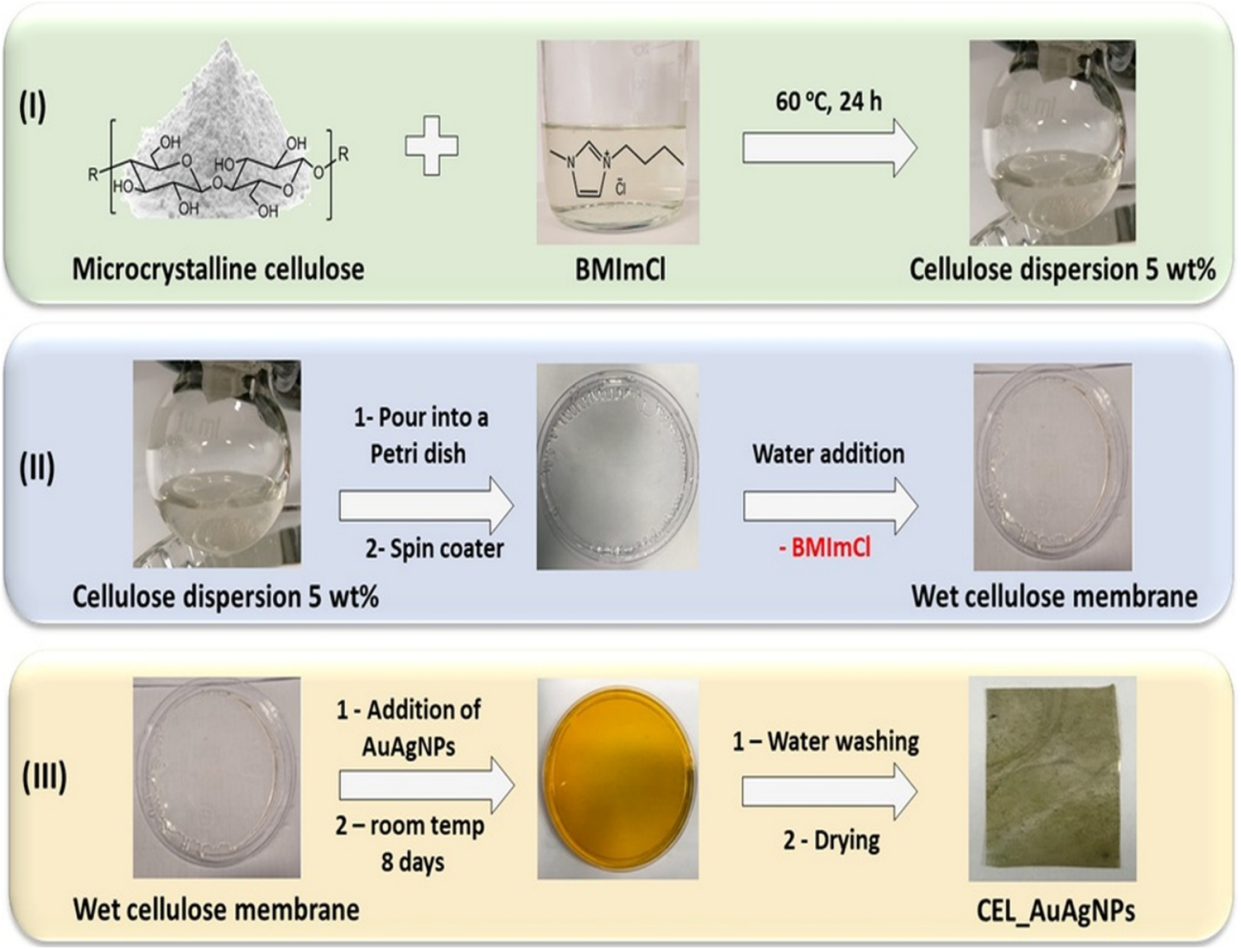
CEL_AuAgNPs membrane preparation scheme.

Preparing cellulose membranes decorated with AuAgNPs involves three critical steps, as illustrated in Figure 3. **Step I. Dissolution of cellulose in an ionic liquid (BMImCl):** The ionic liquid BMImCl effectively disrupts cellulose’s strong hydrogen bond network, making it easily processable for shaping into membranes or other forms. We dissolved microcrystalline cellulose in BMImCl at a concentration of 5 wt % **Step II. Preparation of the cellulose membrane:** This pivotal step involves the regeneration method, a well-established procedure within our research group.^36,42,43^ The resulting membrane has excellent mechanical properties and is insoluble in water and common solvents, guaranteeing durability and versatility. **Step III. Impregnation of AuAgNPs onto the cellulose membrane:** We uniformly integrate bimetallic nanoparticles into the membrane in this innovative approach. A specific amount of AuAgNPs dispersion was introduced into the wet cellulose membrane, and the system was allowed to remain in contact for a defined period.

**Figure 4:**
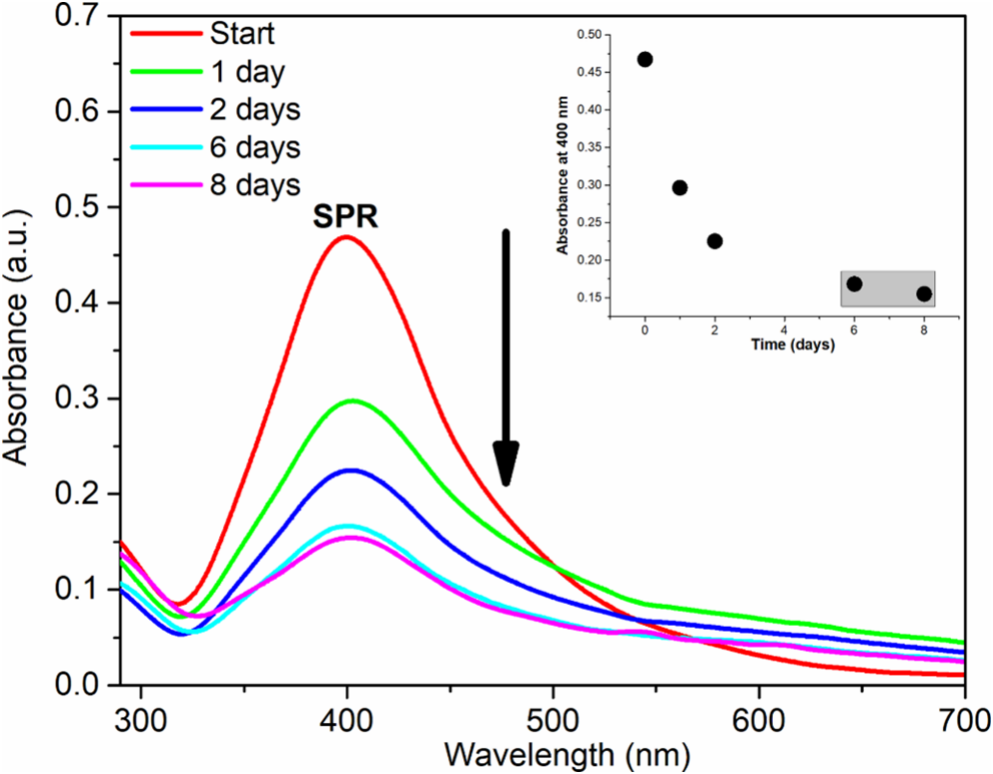
UV–vis of aliquots of the dispersion during impregnation of AuAgNPs into the cellulose membrane. Inset: Graph of absorbance at 400 nm versus time.

To assess the impregnation of AuAgNPs into the cellulose membrane, we monitored the process using UV–vis spectroscopy by analyzing aliquots of the dispersion at different time intervals (Figure 4).

We observed a consistent decrease in the intensity of the SPR band of AuAgNPs over time, indicating the gradual impregnation of nanoparticles into the cellulose membrane. This decrease persisted after 6 days, as shown in the inset of Figure 4. Therefore, we selected an 8-day process duration to guarantee the maximum impregnation of AuAgNPs.

**Figure 5:**
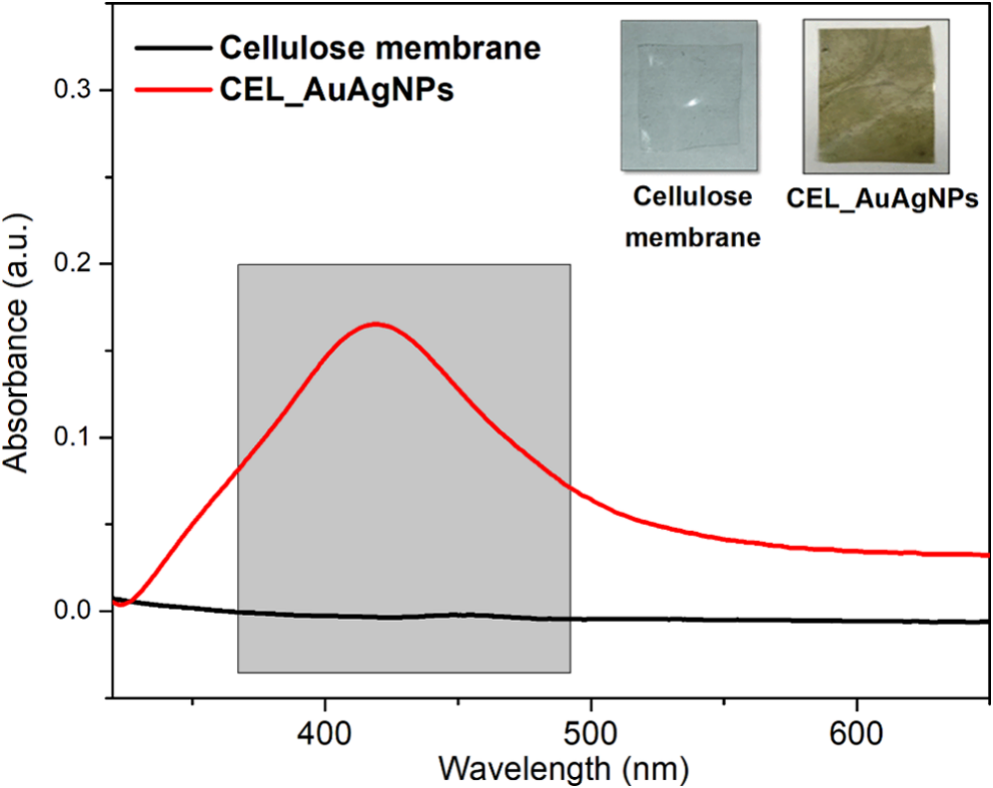
UV–vis of cellulose membrane and CEL_AuAgNPs. Inset: photos of the membranes.

Figure 5 presents the UV–vis spectrum of the CEL_AuAgNPs membrane (red curve) compared to the cellulose membrane (black curve). The inset includes photographs of these membranes. Notably, the color of the membrane changed from colorless to dark yellow after impregnation with nanoparticles, confirming the successful incorporation of AuAgNPs, as also evident in the UV–vis spectra. While the cellulose membrane exhibited no bands in the visible range, the CEL_AuAgNPs spectrum displayed a distinct band around 420 nm, attributed to the SPR band of AuAgNPs. This observation confirmed the presence of bimetallic nanoparticles within the membrane. A short redshift of the SPR maximum from 404 to 420 nm was observed compared to the UV–vis spectrum of the AuAgNPs dispersion (Figure 1C). The reason is that the surface properties influence the SPR, so it changes if the nanoparticles are in dispersion or immobilized in a solid matrix.

**Figure 6:**
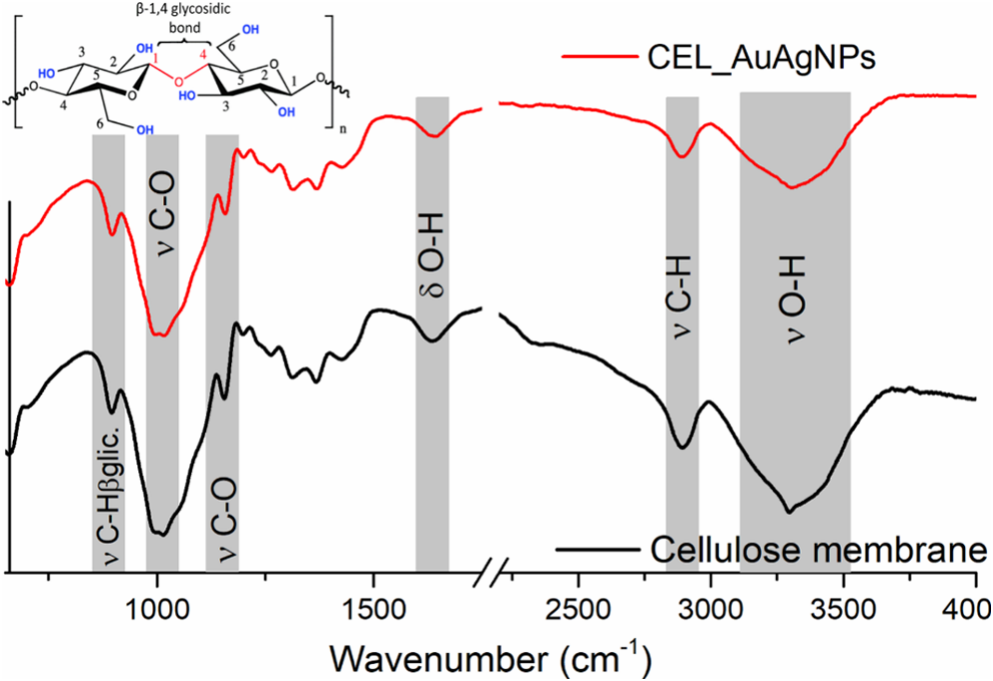
FTIR of cellulose membrane and CEL_AuAgNPs. Inset: chemical structure of cellulose.

Figure 6 presents the Fourier-transformed infrared absorption (FTIR) spectra of both the cellulose and CEL_AuAgNPs membranes. In both spectra, characteristic bands corresponding to cellulose vibration modes (highlighted within gray rectangles) are evident. They include bands at 3328 and 1632 cm^–1^, attributed to OH stretching and bending vibrations, and the band at 2880 cm^–1^ associated with CH stretching mode. The region between 1000 and 1160 cm^–1^ indicates C–O stretching vibrations from primary and secondary alcohols in the cellulose structure. In addition, the sharp band at 900 cm^–1^ is attributed to the skeletal mode vibration at the 1,4-β-glycosidic linkages.^20^ All of these observed bands align with the amorphous cellulose structure (type II) obtained after the dissolution/regeneration of microcrystalline cellulose, as reported in previous studies.^44^ Notably, no significant differences were identified upon comparing the FTIR spectrum of the cellulose membrane with that of the CEL_AuAgNPs membrane. This finding suggests that the chemical integrity of cellulose remained unaltered after the impregnation of AuAgNPs, which is a beneficial aspect.

**Figure 7:**
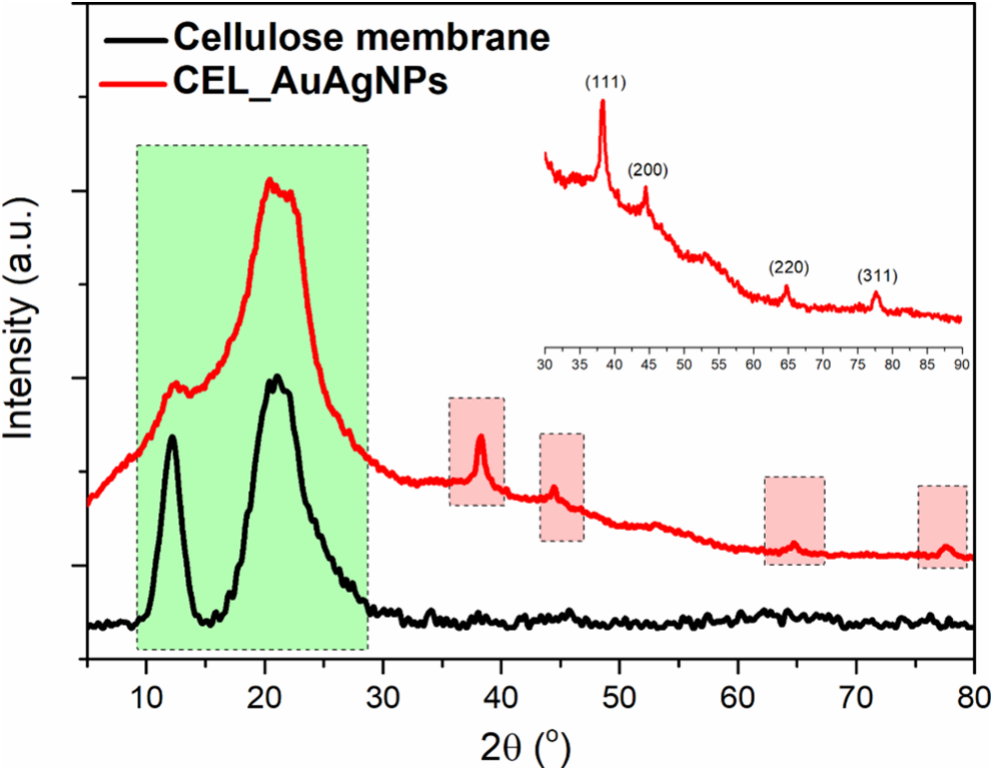
XRD of cellulose membrane and CEL_AuAgNPs.

Figure 7 displays the X-ray diffractograms (XRD) for both CEL_AuAgNPs (in red) and the pristine cellulose membrane (in black). Both diffractograms exhibit prominent halos at 12 and 20° (highlighted within the green rectangle), characteristic of cellulose type II (amorphous), consistent with the FTIR results.^44^ Notably, the CEL_AuAgNPs diffractogram shows a visible reduction in the intensity of the peak at 12°, indicating a decrease in cellulose crystallinity following the addition of nanoparticles. Additionally, in the CEL_AuAgNPs diffractogram, distinct peaks are evident at 2θ angles of 38, 44, 66, and 77° (highlighted within the red rectangles). These correspond to the (111), (200), (220), and (311) planes of the face-centered cubic phase of both Au and Ag, with very similar lattice constants (JCPDS: 4-0783 and 4-0784, respectively).

**Figure 8:**
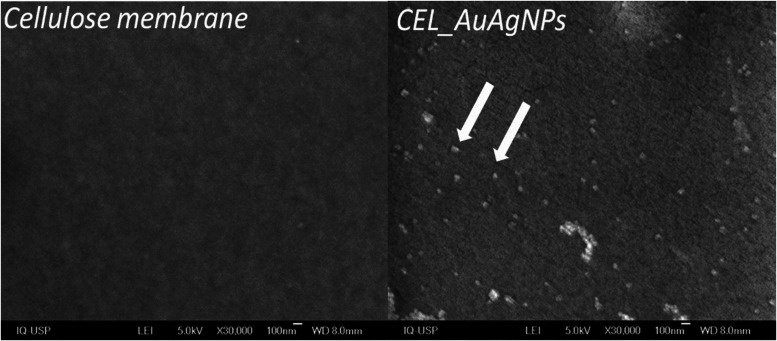
SEM images of cellulose membrane and CEL_AuAgNPs.

To further assess the presence and distribution of AuAgNPs within the membrane, we conducted scanning electron microscopy (SEM) imaging, as depicted in Figure 8. The images reveal a surface that maintains the smooth and uniform characteristics typically seen in regenerated cellulose membranes.^45,46^ However, the difference in the CEL_AuAgNPs membrane is the discernible presence of lighter particles, indicated by white arrows, which correspond to the AuAgNPs. These particles exhibit a homogeneous distribution throughout the entire membrane.

**Figure 9:**
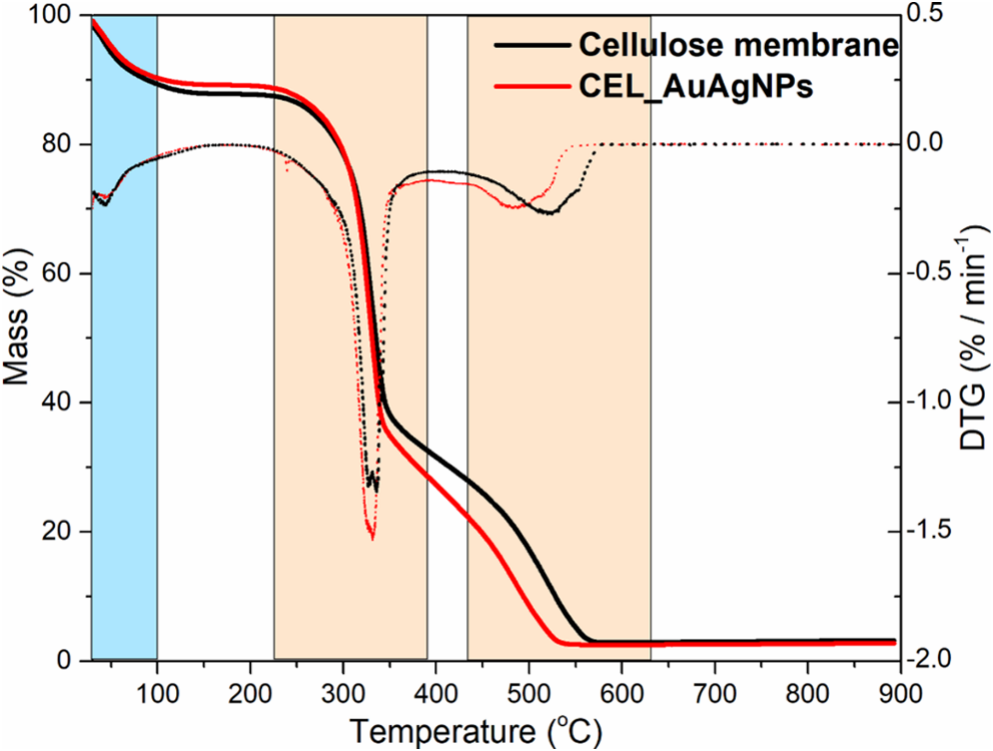
TGA of cellulose membrane and CEL_AuAgNPs. Alumina crucibles, airflow (100 mL/min), and heating rate 10 °C/min.

Thermogravimetric analysis (TGA) was conducted to assess the thermal properties of the membranes (Figure 9). The TG curves for both membranes describe three primary stages of weight loss, highlighted in different colors. The initial decrease in weight from room temperature to 100 °C can be attributed to the expulsion of adsorbed water, accounting for approximately 10% of the total weight loss. Subsequent stages, observed within the temperature range of 240 to 600 °C, indicate the thermal decomposition of cellulose. Both membranes exhibited a similar degradation onset at approximately 250 °C, indicating their notable thermal stability. This characteristic is essential in applications such as catalysis and other fields requiring high-temperature stability.

The metal content within the membranes was quantified using inductively coupled plasma-optical emission spectroscopy (ICP-OES). Specifically, the CEL_AuAgNPs membrane contained approximately 0.339% silver (Ag) and 0.069% gold (Au), resulting in an Ag/Au molar ratio of 8.8. This molar ratio suggests a more substantial silver shell, which aligns with the findings obtained from the UV–vis analysis.

**Figure 10:**
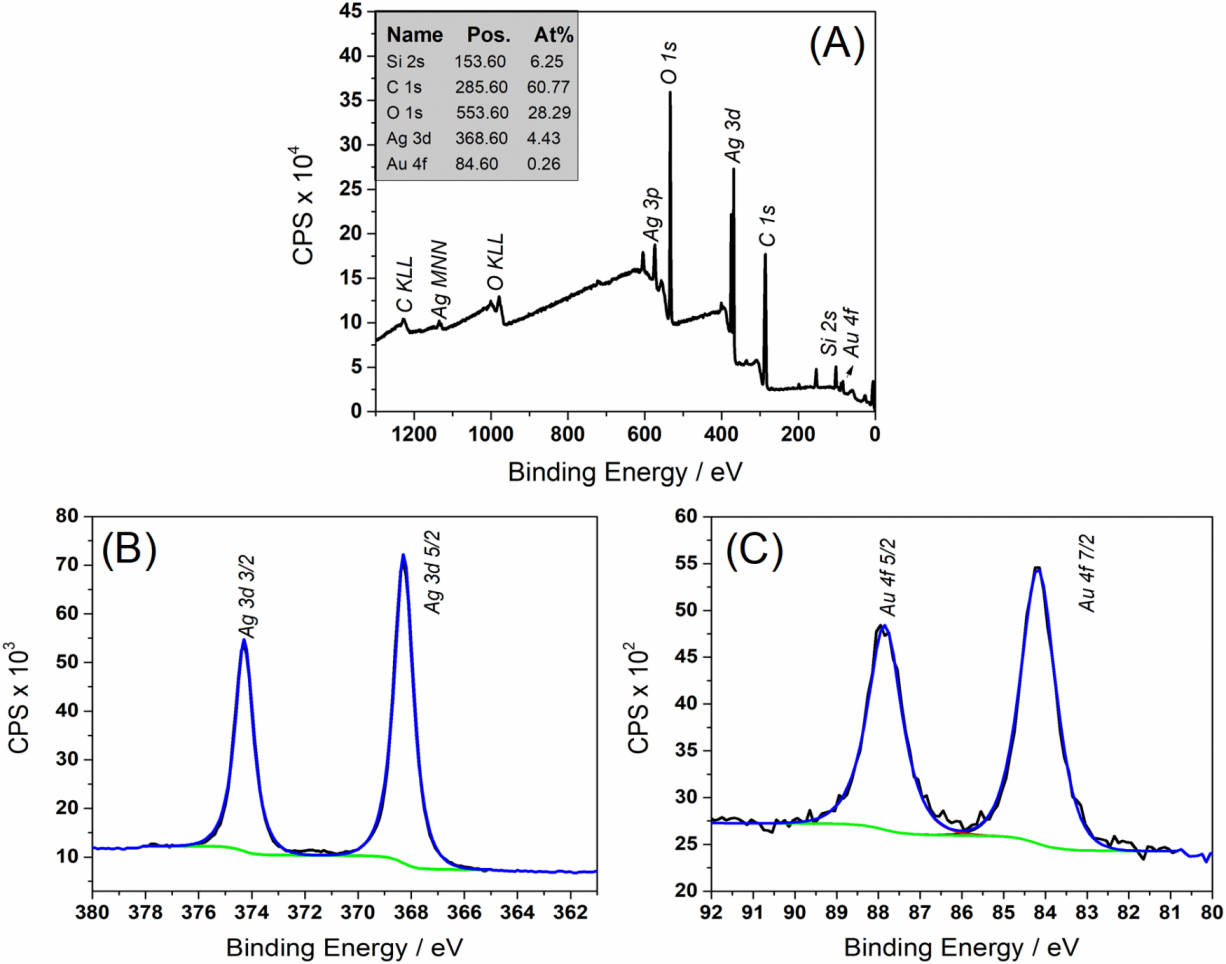
(A) Survey spectrum (B) Ag 3d (C) Au 4f core level XPS spectra for the CEL_AuAgNPs membrane.

X-ray Photoelectron Spectroscopy (XPS) was utilized to analyze the surface chemical composition of the CEL_AuAgNP sample. The full XPS spectra (Figure 10A) predominantly revealed four peaks corresponding to carbon, oxygen, silver, and gold, confirming the presence of cellulose and these metals on the surface. The atomic percent of silver was quantified at 4.43%, significantly higher than gold’s (0.26 atom %). This disparity in atomic percentage between silver and gold can be attributed to the synthesis process of the bimetallic nanoparticles, which likely favored a thicker silver coating on the gold nanoparticle seeds, which agrees with the findings obtained by UV–vis and TEM analyses. The silver peaks at 368.3 eV (Ag 3d_5_/_2_) and 374.3 eV (Ag 3d_3_/_2_) showed a spin–orbit separation of approximately 6 eV, indicative of metallic silver (Figure 10B). The Au 4f spectrum displayed characteristic peaks at 84.2 eV (Au 4f_7_/_2_) and 87.9 eV (Au 4f_5_/_2_), with a spin–orbit split of about 3.7 eV, consistent with metallic gold (Figure 10C).

The catalytic efficiency of the membrane containing bimetallic nanoparticles was evaluated by reducing 4-nitrophenol (4-NP) to 4-aminophenol (4-AP) using sodium borohydride as the reducing agent. This reaction serves as a widely recognized model for evaluating catalyst performance.^47,48^ It offers the advantage of easy assessment of catalytic activity through continuous monitoring via UV–vis spectroscopy over time, eliminating the need for product isolation.^49,50^ It is worth noting that the knowledge of how to convert a nitro group effectively and efficiently to an amino group can lead to advancements in drug synthesis and the development of new materials.

**Figure 11:**
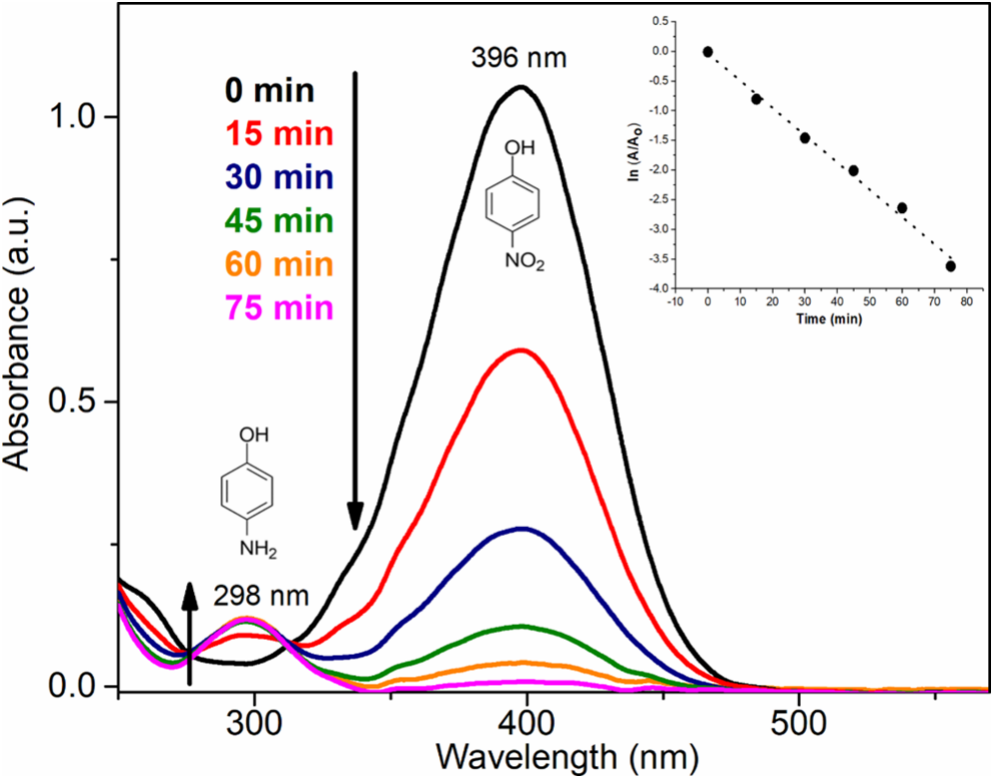
UV–vis spectra from the catalytic reduction experiment using CEL_AuAgNPs.

At room temperature, the catalytic experiments were conducted in aqueous media containing 4-NP (3.00 mM), NaBH_4_ (300 mM), and a 10.0 mg piece of the CEL_AuAgNPs membrane. Aliquots of the reaction mixture were periodically collected and subjected to UV–vis analysis (Figure 11). A 100-fold excess of the reducing agent was employed to maintain a pseudo-first-order condition for the kinetics evaluation.^51^

Upon the addition of the catalyst, a noticeable color change in the solution was observed over time, shifting from yellow to colorless. This color change indicates the reduction of 4-NP, a confirmation validated by UV–vis analysis. Figure 11 shows that the characteristic 4-NP band at 396 nm diminishes with time, accompanied by the appearance of a new band at 298 nm, which is typical of 4-AP. The conversion of 4-NP reached 90% within 52 min and achieved complete conversion after 75 min. From this data, the natural logarithm of the ratio (ln(*A*/*A*_0_)) was computed, with *A*_0_ representing the initial absorbance and *A* the absorbance at a specific time point. The plot of ln(*A*/*A*_0_) versus time (inset of Figure 11) yielded a linear relationship, indicative of first-order kinetics governing the reduction of 4-NP. The apparent rate constant (*k*_app_) for this catalytic process was calculated to be 0.04591 min^–1^, and the turnover frequency (TOF) value for the reduction of 4-NP was calculated as 394 h^–1^.

An experiment using a pure cellulose membrane was conducted, and no reaction occurred even after 24 h, emphasizing that the reaction was catalyzed by the bimetallic nanoparticles supported on the cellulosic substrate.

**Figure 12:**
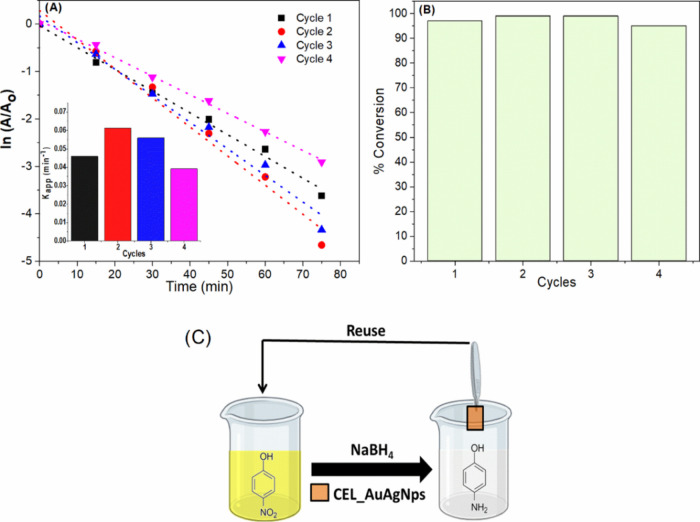
Cyclability experiments: (A) ln(*A*/*A*_0_) versus time, inset *K*_app_ of different cycles and (B) % conversion of each cycle, and (C) experimental procedure scheme.

To evaluate the reusability of the CEL_AuAgNPs, we subjected it to a series of consecutive 4-nitrophenol reduction reactions. In each cycle, the membrane was removed from the reaction solution using a tweezer, followed by a thorough rinse with distilled water before being employed in a new 4-NP solution, as illustrated in Figure 12C. Since the initial experiment achieved the highest conversion rate within 75 min, all subsequent cycles were performed with the same duration. Figure 12A displays plots of ln(*A*/*A*_0_) against time for all cycles and the *k*_app_ in the inset. The higher constant rate in cycle 2 in comparison with cycle 1 can be attributed to two factors: (1) the hydrophilicity of the cellulose membrane, which requires an initial hydration or conditioning phase during the first cycle, stabilizing in subsequent uses; and (2) the incomplete adsorption equilibrium between the catalytic nanoparticles and 4-NP during the first cycle, which becomes fully established in later cycles.^52^ These factors lead to improved catalytic performance after the initial cycle. Then, it is possible to observe a slight decrease in the constant rate after each cycle. However, the maximum conversion of 4-NP (95%) was achieved in all experiments (Figure 12B). These experiments demonstrated that the CEL_AuAgNPs membrane maintains its catalytic efficiency over the multiple tested cycles. One of the fundamental advantages of this catalyst is its ease of removal from the reaction medium and subsequent reuse. Unlike powdered or dispersed nanoparticles, the membrane eliminates the need for additional isolation procedures such as filtration or centrifugation.

To further evaluate the stability of the CEL_AuAgNPs sample after catalysis, FTIR analysis was conducted on the membrane after multiple cycles (Figure S4). The results confirmed that there were no significant changes in its chemical composition, demonstrating the stability and durability of the sample.

The direct comparison of our results with existing literature was hindered by the absence of studies involving AuAg bimetallic nanoparticles supported on unmodified cellulose to reduce 4-nitrophenol. So, we compared our bimetallic catalyst with the monometallic counterparts, which were also supported on an unmodified cellulose membrane. In this comparison, the amounts of 4-NP and catalyst were identical. The TOF value for the cellulose membrane containing gold nanoparticles was 137 h^–1^,^36^ and the one with silver was 290 h^–1^.^43^ In comparison, our bimetallic catalyst presented a TOF of 394 h^–1^, demonstrating an enhancement in catalytic performance. These results highlight the synergistic effect between the gold and silver in the core–shell structure, confirming its superior catalytic efficiency compared to the monometallic systems. The synergistic effects of the Au–Ag interface enhance catalytic efficiency by improving hot electron generation and transfer while combining the stability of gold with the high intrinsic catalytic activity of silver for 4-NP reduction. The presence of the gold core and silver shell creates a bimetallic interface, where the difference in Fermi energy levels generates contact potential. This potential drives the efficient transfer of hot electrons from the gold core to the silver shell, ensuring a continuous flow of electrons to the reaction site. This mechanism minimizes electron–hole recombination, a major limitation in monometallic silver nanoparticles, and enhances the availability of hot electrons required for the reduction of 4-NP to 4-AP.^53^ Additionally, the thick silver shell in the bimetallic nanoparticle is an active surface for 4-NP adsorption and reduction.

## 4 Conclusions

This study introduces a novel approach for synthesizing a heterogeneous catalyst based on unmodified cellulose membranes containing bimetallic gold–silver nanoparticles (AuAgNPs). The AuAg nanoparticles, with a core–shell structure, were successfully embedded within the cellulose membranes without altering their chemical composition or amorphous nature, as confirmed by characterization techniques. Microscopic images demonstrated a uniform distribution of AuAgNPs throughout the membrane. Thermal analysis revealed substantial stability, a critical attribute for a wide range of applications. The catalytic efficiency of the CEL_AuAgNPs membrane was demonstrated in the reduction of 4-nitrophenol to 4-aminophenol, a model reaction for evaluating catalyst performance. This membrane exhibited exceptional catalytic activity, following first-order kinetics. Furthermore, it maintained its effectiveness across multiple uses, highlighting its reusability and practicality in heterogeneous catalysis. This catalyst displayed a significantly higher TOF than cellulose membranes containing only gold or silver nanoparticles, emphasizing its superior performance over monometallic counterparts. These findings advance the fields of catalysis and materials science and pave the way for developing efficient, stable, and recyclable catalysts.

## Supporting Information Available

TEM of AuNPs dispersion (Figure S1), EDX-line scan for the AuAgNPs dispersion (Figure S2), DLS analysis for the AuAgNPs sample (Figure S3), and FTIR of the CEL_AuAgNPs membrane after catalytic experiments (Figure S4) (PDF)
