# Supplementary material for: Cellulose Membranes Embedded with Gold–Silver Bimetallic Nanoparticles for the Efficient Reduction of 4-Nitrophenol
Source: ACS Omega. 2025 Apr 7;10(15):14805–15. doi: 10.1021/acsomega.4c09636 (PMC12019471; doi:10.1021/acsomega.4c09636)
Supplement: Supplementary file 1 — ao4c09636_si_001.pdf [file ao4c09636_si_001.pdf]

Supporting Information

Cellulose Membranes Embedded with Gold-Silver Bimetallic Nanoparticles for Efficient Reduction of 4-Nitrophenol

Maíra Vasconcelos de Carvalho,[1] João Henrique G. Lago,[2] Samar Hajjar-Garreau,[3,4] Fernanda F. Camilo[1*], Larissa V. F. Oliveira[2*]

[1]*Chemistry Department, Institute of Environmental, Chemical and Pharmaceutical Sciences, Federal University of São Paulo, SP-09913-030, Diadema, Brazil*

[2]*Center of Natural Sciences and Humanities, Federal University of ABC, SP-09210-580, Santo Andre, Brazil*

[3]*Institut de Science des Matériaux de Mulhouse, CNRS UMR 7361, Université de Haute-Alsace, F-68100 Mulhouse, France*

[4]*Université de Strasbourg, F-67081 Strasbourg, France*

* *Corresponding author*: Larissa Verena Figueiredo de Oliveira
Centro de Ciências Naturais e Humanas (CCNH/UFABC), Universidade Federal do ABC.
Avenida dos Estados, 5001, CEP: 09210580, Santo André- SP, Brasil
E-mail: larissa.verena@ufabc.edu.br

* *Corresponding author*: Fernanda Ferraz Camilo
Laboratório de Materiais Híbridos, Instituto de Ciências Ambientais, Químicas e Farmacêuticas, Universidade Federal de São Paulo.
Rua São Nicolau, 210, CEP: 09913-030, Diadema – SP, Brasil
E-mail: ffcamilo@unifesp.br

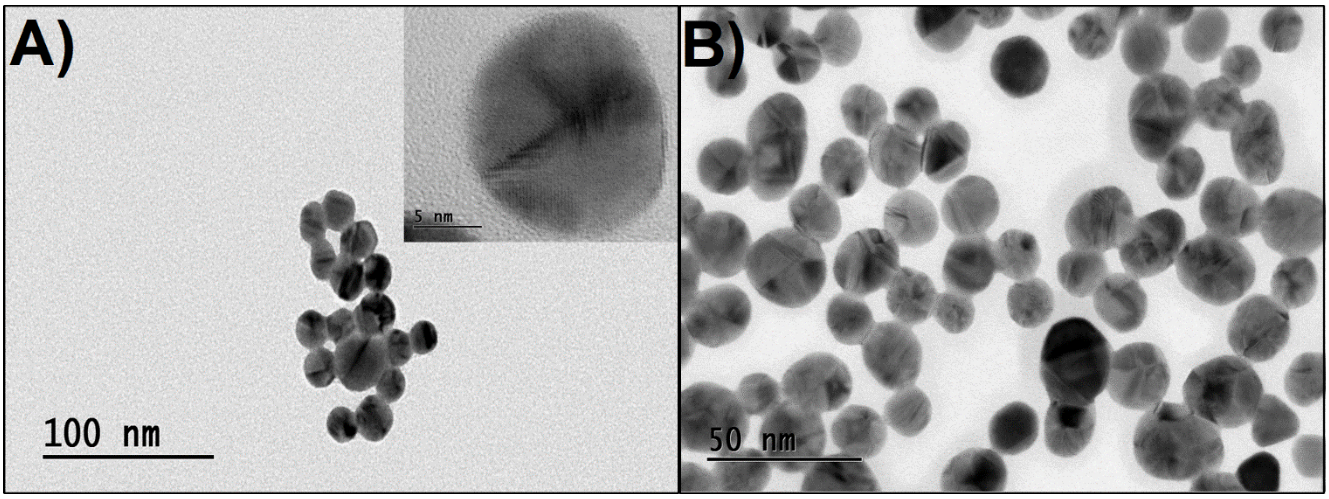

Figure S1: TEM images of AuNPs dispersion at different magnifications

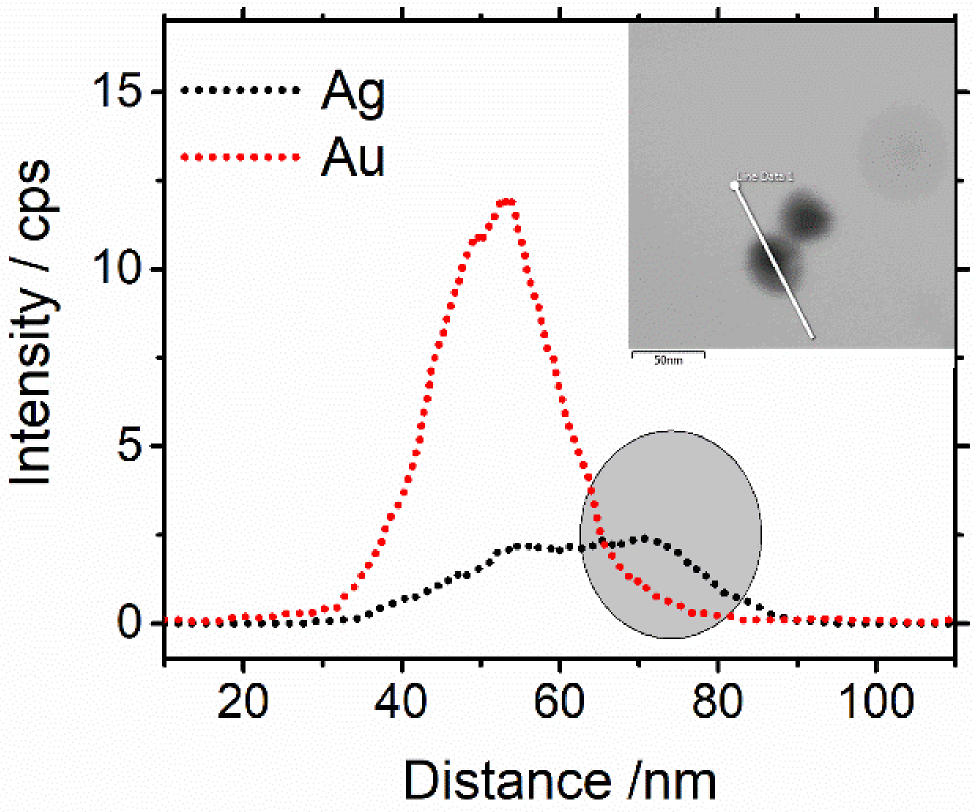

Figure S2: EDX line-scan analysis of AuAgNPs dispersion. Inset: STEM image with the corresponding line-scan location

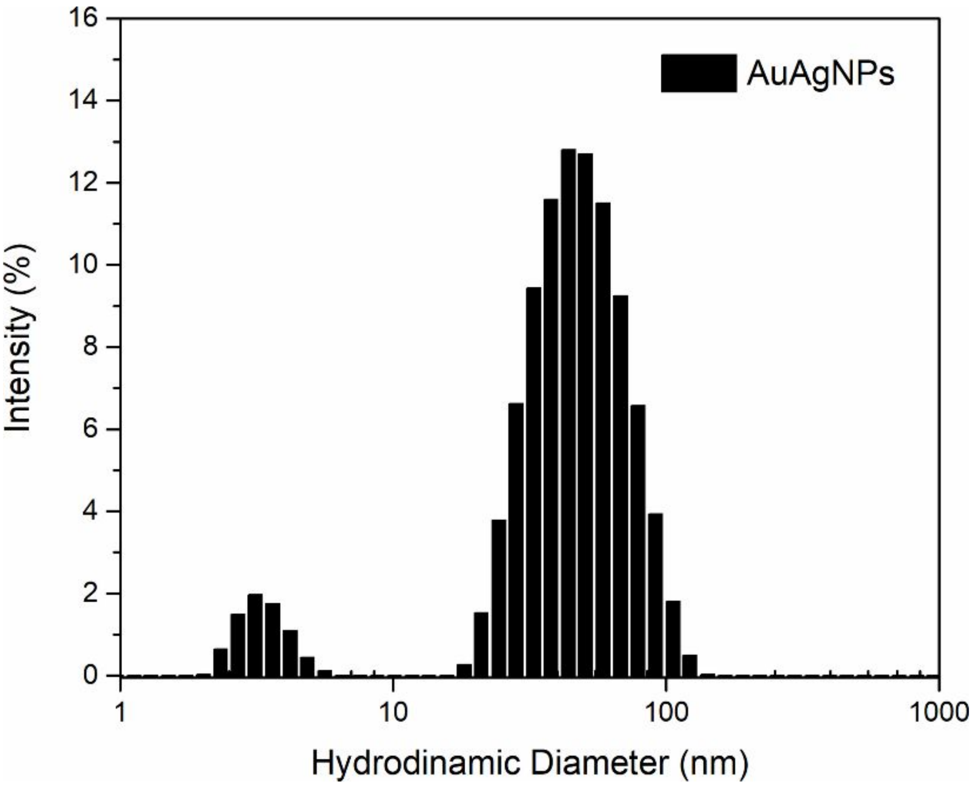

Figure S3: Scattered light intensity vs hydrodynamic diameter (Dh) for AuAgNPs sample

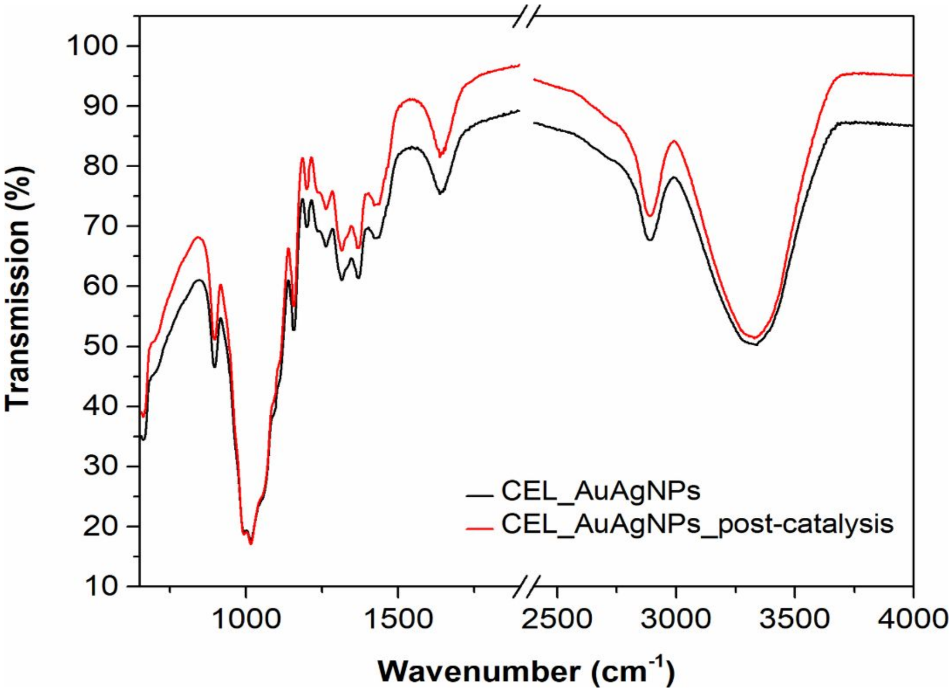

Figure S4: FTIR analysis of the CEL_AuAgNPs membrane before and after multiple cycles of catalysis
